# Prevalence of hyperuricemia among type 2 diabetes mellitus patients in Africa: a systematic review and meta-analysis

**DOI:** 10.1186/s12902-023-01408-0

**Published:** 2023-07-18

**Authors:** Ermiyas Alemayehu, Temesgen Fiseha, Getachew Mesfin Bambo, Samuel Sahile Kebede, Habtye Bisetegn, Mihret Tilahun, Habtu Debash, Hussen Ebrahim, Ousman Mohammed, Melaku Ashagrie Belete, Alemu Gedefie

**Affiliations:** 1 Department of Medical Laboratory Sciences, College of Medicine and Health Sciences, Wollo University, PO.Box 1145, Dessie, Ethiopia; 2 Department of Medical Laboratory Sciences, College of Health Sciences, Mizan-Tepi University, PO.Box 260, Mizan, Ethiopia

**Keywords:** Prevalence, Hyperuricemia, Type 2 diabetes, Africa, Systematic review, Meta-analysis

## Abstract

**Background:**

Hyperuricemia increases morbidity and mortality in type 2 diabetic individuals. It is linked to the expansion of diabetes and cardiovascular diseases indicators, as well as being a significant predictor of coronary artery disease. It also leads to a poor prognosis and increment of diabetic complications including diabetic neuropathy, retinopathy, and nephropathy. Therefore, this systematic review and meta-analysis was aimed to determine the pooled prevalence of hyperuricemia among type 2 diabetes mellitus patients in Africa.

**Methods:**

We conducted a systematic review and meta-analysis following the Preferred Reporting Items for Systematic Reviews and Meta-Analysis guidelines. To identify relevant articles, we searched electronic databases such as PubMed, Google Scholar, African Journal Online, Science Direct, Embase, ResearchGate, Scopus, and Web of Sciences. The quality of the included studies was assessed using the Newcastle-Ottawa Quality Assessment Scale. Statistical analysis was performed using Stata 14.0 software. To evaluate heterogeneity, we utilized Cochran’s Q test and I^2^ statistics. Publication bias was assessed through the examination of a funnel plot and Egger’s test. The pooled prevalence was estimated using a random effect model. Furthermore, sub-group and sensitivity analyses were conducted.

**Results:**

The overall pooled prevalence of hyperuricemia among type 2 diabetic patients in Africa was 27.28% (95% CI: 23.07, 31.49). The prevalence was highest in Central Africa 33.72% (95% CI: 23.49, 43.95), and lowest in North Africa 24.72% (95% CI: 14.38, 35.07). Regarding sex, the pooled prevalence of hyperuricemia among female and male type 2 diabetic patients was 28.02% (95% CI: 22.92, 33.48) and 28.20% (95% CI: 22.92, 33.48), respectively.

**Conclusion:**

This systematic review and meta-analysis showed a high prevalence of hyperuricemia among type 2 diabetic patients. So, regular screening and diagnosis of hyperuricemia required for preventing its pathological effects and contribution to chronic complications of diabetes.

**Systematic review registration:**

: PROSPERO (2022: CRD42022331279).

**Supplementary Information:**

The online version contains supplementary material available at 10.1186/s12902-023-01408-0.

## Introduction

Diabetes mellitus (DM) is a group of heterogeneous disorders with multiple etiologies characterized by a chronic hyperglycemia resulting from defects in the insulin secretion and/or insulin action [1]. According to the current classification, diabetes mellitus is classified as type 1 diabetes (T1DM) and type 2 diabetes (T2DM) [2]. It hurts people’s functional abilities and quality of life, resulting in severe morbidity and mortality [3]. It is one of the world’s most serious public health issues, imposing a significant threat to public health and socioeconomic development [4]. According to International Diabetic Federation, worldwide, diabetes affects an estimated 537 million adults. Approximately 6.7 million adults are estimated to have died as a result of diabetes, or its complications in 2021. In African region an estimated 24 million individuals were affected by diabetes and 416,000 deaths occurred in 2021[5].

Type 2 diabetes is characterized by impaired insulin secretion by pancreatic cells or a failure of tissues to respond to insulin [6]. It is the most common type of diabetes accounting for over 90% of all diabetes cases worldwide [5]. It is also a critical public health concern that has a significant impact on human life and healthcare costs [7].

Hyperuricemia is a condition in which a person’s serum uric acid (UA) level is abnormally high. In normal metabolic processes, UA is a byproduct of the breakdown and metabolism of purine substances [8]. It is an antioxidant that helps to protect atherosclerosis in its initial phases. When its level rises in later phases of atherosclerosis, it works as a pro-oxidant rather than an antioxidant [9]. Humans are more likely to be exposed to hyperuricemia than other mammals due to lack of the enzyme urate oxidase results from genetic alteration that breaks down uric acid [10]. In diabetic individuals, it increases morbidity and mortality. It also leads to a poor prognosis and increment of diabetic complications including diabetic neuropathy, retinopathy, and nephropathy [11]. Through atherosclerotic mechanisms, it has been also linked to several cardiovascular diseases (CVD) [12, 13].

In T2DM patients, it is linked to the expansion of diabetes and CVD indicators, as well as being a significant predictor of coronary artery disease [14, 15]. There is also a strong association between plasma UA concentrations and glucose consumption in T2DM [16]. According to a prospective study, a high amount of serum UA has been linked to the onset of T2DM [17]. Recently, serum UA level has attracted interest as a potential biomarker-dependent predictor of high blood pressure, diabetes mellitus, and chronic kidney disease [18].

Hyperuricemia has several side effects that have been linked to diabetic nephropathy. Endothelial dysfunction, enhanced renin-angiotensin-aldosterone system activity, and stimulation of inflammatory pathways, as well as pro-fibrotic cytokine activation [19–21], have been shown to contribute to the advancement of micro vascular disease and hence renal damage in diabetic nephropathy. Therefore, this systematic review and meta-analysis was aimed to determine the pooled prevalence of hyperuricemia among type 2 diabetes mellitus patients in Africa.

## Methods

### Design and registration

This systematic review and meta-analysis was conducted in compliance with the guidelines provided by Preferred Reporting Items for Systematic Reviews and Meta-Analyses (PRISMA) [22]. The focus of this systematic review and meta-analysis was studies targeting on hyperuricemia among individuals with type 2 diabetes mellitus in Africa. The protocol for this study was registered at the International Prospective Register of Systematic Reviews (PROSPERO) under the registration number CRD42022331279.

### Source of data and search strategies

To ensure a thorough investigation, a team of four reviewers (EA, AG, GM, and SS) conducted an extensive literature search. Major electronic databases such as PubMed, Google Scholar, African Journal Online, Science Direct, Embase, ResearchGate, Scopus, and Web of Sciences were utilized to locate relevant articles. Additionally, the proceedings of professional associations and university repositories were screened. To include any potentially overlooked studies, a direct Google search was performed using the bibliographies of the identified studies. The search spanned from February 2, 2022, to April 20, 2022.

To optimize the search process, MeSH terms and a combination of key terms derived from the review question were employed. The following key terms were used in various combinations: “hyperuricemia, hyperuricemia, uric acid disorders, serum uric acid level, type 2 diabetes, non-insulin-dependent diabetes, insulin resistance, and Africa.“ These key terms were used individually as well as in conjunction with the Boolean operators “OR” and “AND” as necessary. Furthermore, the search was expanded by combining the aforementioned search terms with the names of all African countries.

### Eligibility criteria

#### Inclusion criteria

The final meta-analysis included observational studies conducted in Africa that met specific criteria and reported the prevalence of hyperuricemia among individuals with type 2 diabetes mellitus (T2DM). This systematic review and meta-analysis included original articles published in peer-reviewed journals or grey literature, specifically those published in English. The studies needed to report the prevalence of hyperuricemia as their primary outcome, and they were considered up until April 20, 2022.

#### Exclusion criteria

We excluded qualitative research studies, review articles, case reports, narrative reviews, conference abstracts lacking complete information or where authors did not respond to our request for full-text, editorials, commentaries, letters to the editor, author replies, and other publications that did not provide quantitative information on the prevalence of hyperuricemia. Additionally, studies specifically focused on type 1 diabetes mellitus were also excluded.

### Study selection

After conducting searches using electronic databases, conference proceedings, and bibliographic search, the articles were imported into EndNote version 20 software. Duplicates were then eliminated. Two independent reviewers (EA and AG) thoroughly examined the title, abstract, and full-text quality of each selected paper, adhering to the predetermined eligibility criteria. In cases where there was a disagreement between the two reviewers, a third reviewer (TF) was involved in resolving the discrepancy through discussion. The final selection of articles for the review was determined through this collaborative process.

### Data extraction and quality assessment

The selected papers underwent a thorough evaluation, and relevant information was extracted and summarized using a Microsoft Office Excel extraction table. This systematic review and meta-analysis were reported according to the preferred reporting items for systematic review and meta-analysis (PRISMA) guideline [23]. To assess the quality of the included studies, the Newcastle-Ottawa Quality Assessment Scale (NOS) adapted for cross-sectional studies was employed [24]. This tool assigns a maximum of ten stars. Articles were categorized as “very good quality” if they received nine to ten stars or higher, “good quality” for seven to eight stars, “satisfactory quality” for five to six stars, and “unsatisfactory” for zero to four stars.

The prevalence findings regarding hyperuricemia were independently extracted by three reviewers (GMB, NA, and SS). The Microsoft Excel sheet was structured with subheadings agreed upon by all reviewers. The extracted data were meticulously cross-checked by the three reviewers, and any disagreements between the data extractors were resolved through discussions and consensus verification. The data extracted from each study included the first author’s name, publication year, country, sub-region, year of the study, study design, sample size, number of male and female participants, number of positive cases, prevalence of hyperuricemia, and the number of hyperuricemia cases by sex of participants.

### Statistical methods and analysis

The data were extracted into Microsoft Excel and subsequently imported into STATA 14.0 software for statistical analysis. To evaluate the heterogeneity between studies, Cochran’s Q test along with its respective p-value and I^2^ statistics were utilized. Heterogeneity was considered significant when the I^2^ test statistic exceeded 50% [25] and when the Q test and its corresponding p-value were less than 0.05. In line with the protocol, which accounted for potential differences across studies, a random effect model was employed to estimate the pooled prevalence of hyperuricemia among individuals with type 2 diabetes mellitus (T2DM) across multiple studies, providing a 95% confidence interval [26]. The results were presented through a forest plot. The presence of publication bias was indicated by asymmetries observed in the funnel plot and a p-value less than 0.05 from Egger’s test. Subgroup analysis was conducted based on factors such as the participants’ sex, year of publication, sub-regions, and countries where the studies were conducted. Additionally, a sensitivity analysis was performed to assess the impact of individual studies on the overall pooled estimate.

## Results

### Description of included studies

**Fig. 1:**
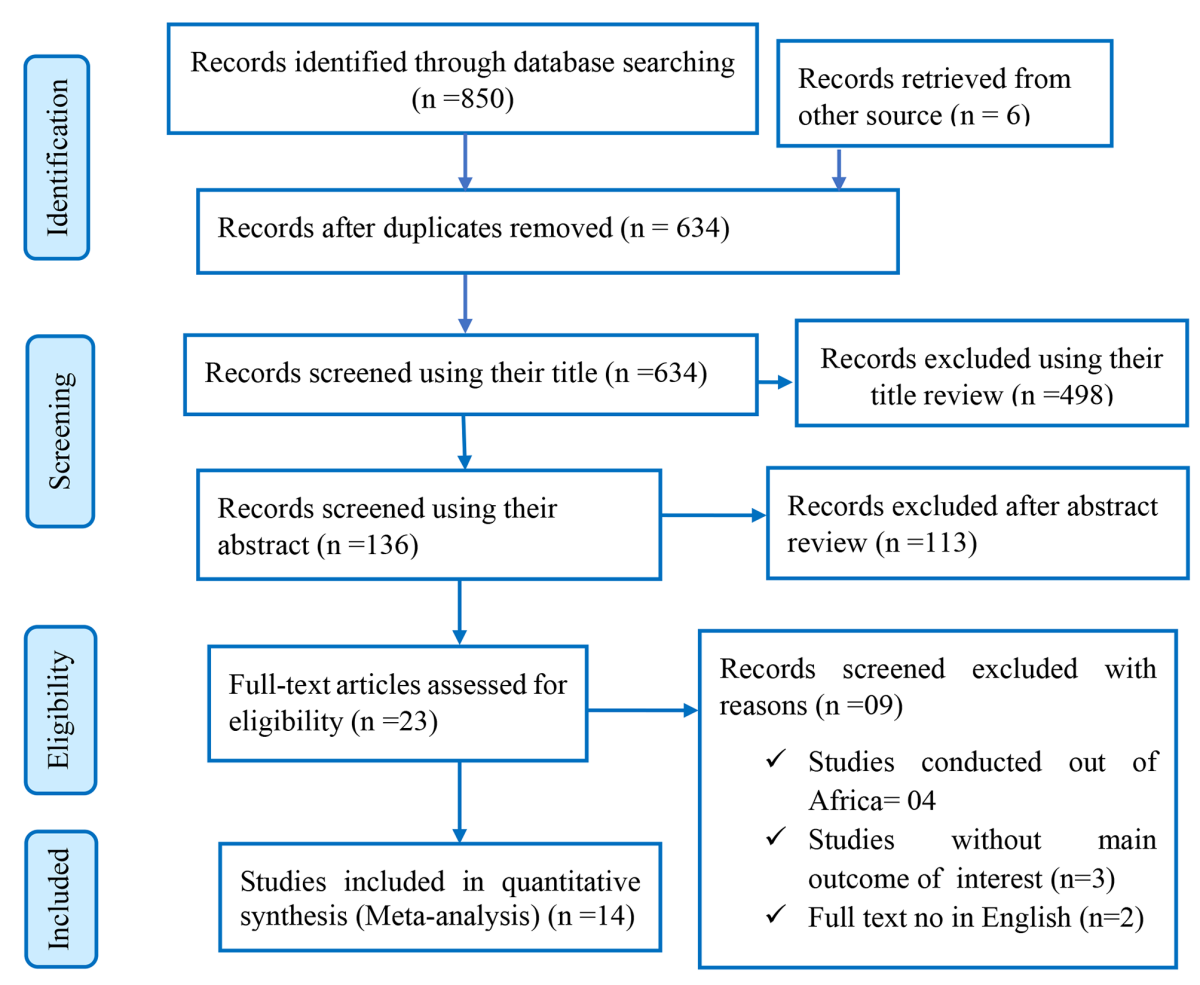
Flow diagram of the included studies for the systematic review and meta-analysis of the prevalence of hyperuricemia in Africa

A total of 856 published articles were accessed through database searches and other sources. 222 articles were removed because of duplication. Then, about 634 articles were screened for their title and abstract, and 611 articles were removed. A total of 23 full-text articles were screened for eligibility criteria. Then, 09 full-text articles were excluded from the analysis for various reasons. Finally, 14 studies were included in this systematic review and meta-analysis for the final analysis (Fig. 1).

### Characteristics of the included studies

A total of 14 original articles conducted in nine African countries were included in this systematic review and meta-analysis. Of these, 4 (28.5%) of them were in East African countries (3 from Ethiopia and 1 from Kenya) [27–30], 4 (28.5%) from West African countries (3 from Nigeria and 1 from Senegal) [31–34], and 3 (21.4%) studies were from North African countries (1 from Egypt, 1 from Sudan, and 1 from Morocco) [35–37]. On the other hand, 2 (14.2%) studies were from only one Central African country (Cameroon) [38, 39], and 1 (7.1%) study was from only one Southern African country (Botswana) [40]. Regarding the study design, the majority (12 studies) were cross-sectional [27–34, 37–40], one was case-control [35], and the other one is a retrospective study [36]. The included articles were a hospital-based study design. A total of 4,060 type 2 diabetic patients were included in this systematic review and meta-analysis. Of these, 2,048 participants were females and the other 2,012 were males. The included studies reported sample sizes that ranged from 80 participants in Cameroon [39] to 736 in Egypt [35]. The highest prevalence of hyperuricemia (45%) was reported from Nigeria in 2014 [31] and the lowermost prevalence (10.7%) was also reported from Nigeria in 2007 [33]. Regarding the quality of the included studies, majority of studies (10 studies) had very good quality and the remaining 4 studies had good quality (Table 1).

**Table 1:** Characteristics of included studies

Authors	Year of publication	Sub-region	County	Study Design	Sample size	Prevalence (%)	SE prevalence	Quality of the study
Arersa et al. [27]	2020	Eastern	Ethiopia	Cross-sectional	287	22	2.44	Very good
Woyesa et al. [28]	2017	Eastern	Ethiopia	Cross-sectional	314	33.8	2.66	Very good
Woldeamlak et al. [29]	2019	Eastern	Ethiopia	Cross-sectional	384	32.2	2.38	Very good
Ogbera et al. [32]	2010	Western	Nigeria	Cross-sectional	603	25	1.76	Very good
Uwakwe et al. [31]	2014	Western	Nigeria	Cross-sectional	100	45	4.97	Good
Akande et al. [33]	2007	Western	Nigeria	Cross-sectional	121	10.7	2.81	Good
Donkeng et al. [39]	2021	Central	Cameroon	Cross-sectional	80	27.5	4.99	Good
Choukem et al. [38]	2016	Central	Cameroon	Cross-sectional	438	38.1	2.32	Very good
Mirghani [37]	2018	Northern	Sudan	Cross-sectional	170	15.3	2.76	Good
Gobusamang et al. [40]	2019	Southern	Botswana	Cross-sectional	334	28	2.45	Very good
Shokat et al. [30]	2019	Eastern	Kenya	Cross-sectional	150	19.3	3.22	Very good
Fennoun et al. [36]	2020	Northern	Morocco	Retrospective	190	26.5	3.20	Very good
Barry et al. [34]	2021	Western	Senegal	Cross-sectional	153	29.4	3.68	Very good
Fouad et al. [35]	2016	Northern	Egypt	Case control	736	32	1.71	Very good

Note: SE; standard error

### Pooled prevalence of hyperuricemia among type 2 diabetes mellitus patients in Africa

**Fig. 2:**
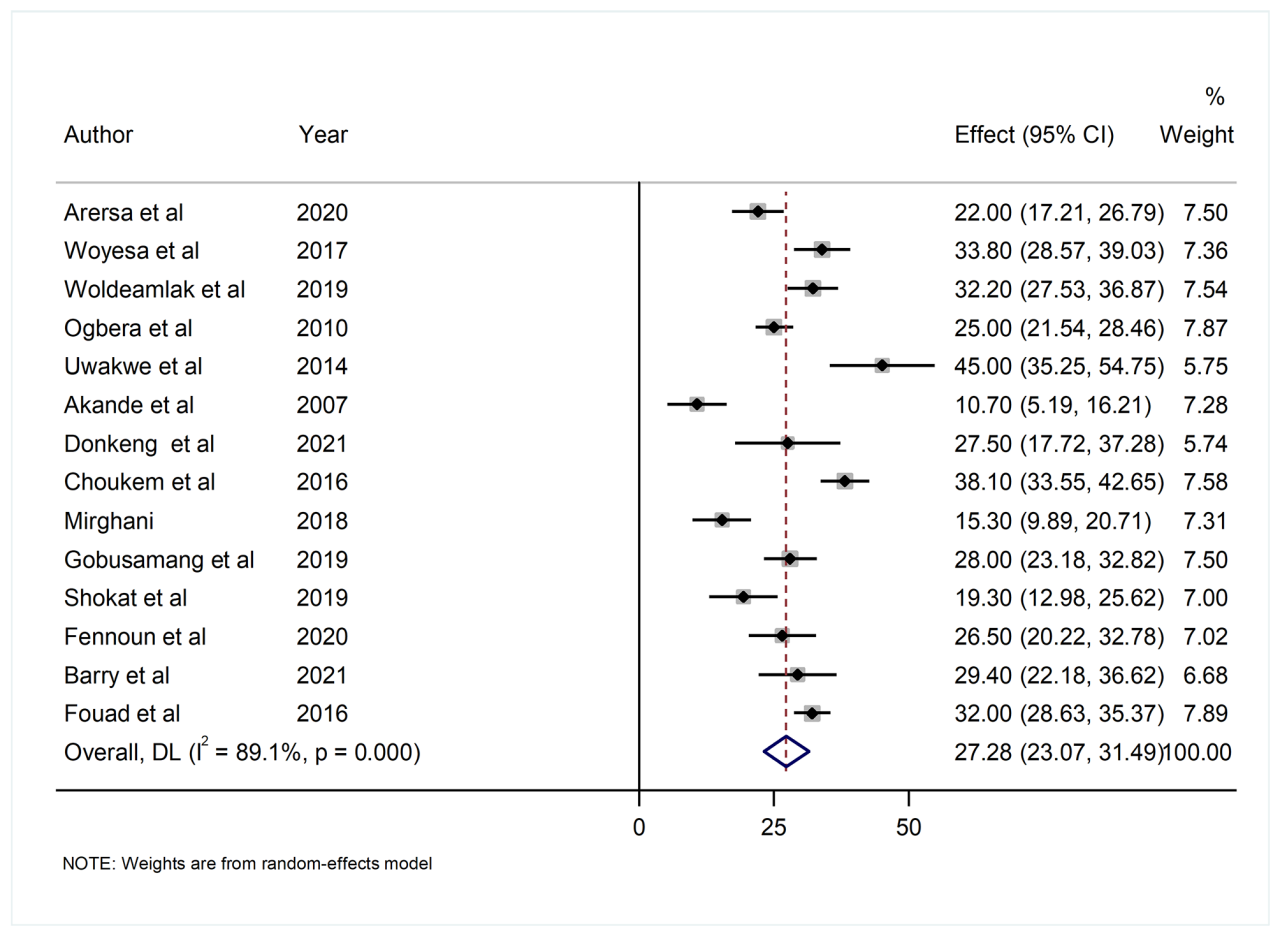
Forest plot showing the pooled prevalence of hyperuricemia among T2DM patients in Africa from random-effect model analysis

The prevalence of hyperuricemia among T2DM patients ranges from 10.7% (95% CI; 5.19–16.21%) to 45% (95% CI; 35.25–54.75%). In random-effect model analysis, the overall pooled prevalence of hyperuricemia among T2DM patients in Africa was 27.28% (95% CI: 23.07, 31.49). There was a high level of heterogeneity with I^2^ value of (89.1%, p < 0.001) and Q test (Tau-squared = 55.62, p < 0.001) (Fig. 2).

### Sub-group analysis

#### Hyperuricemia by sub-region

**Fig. 3:**
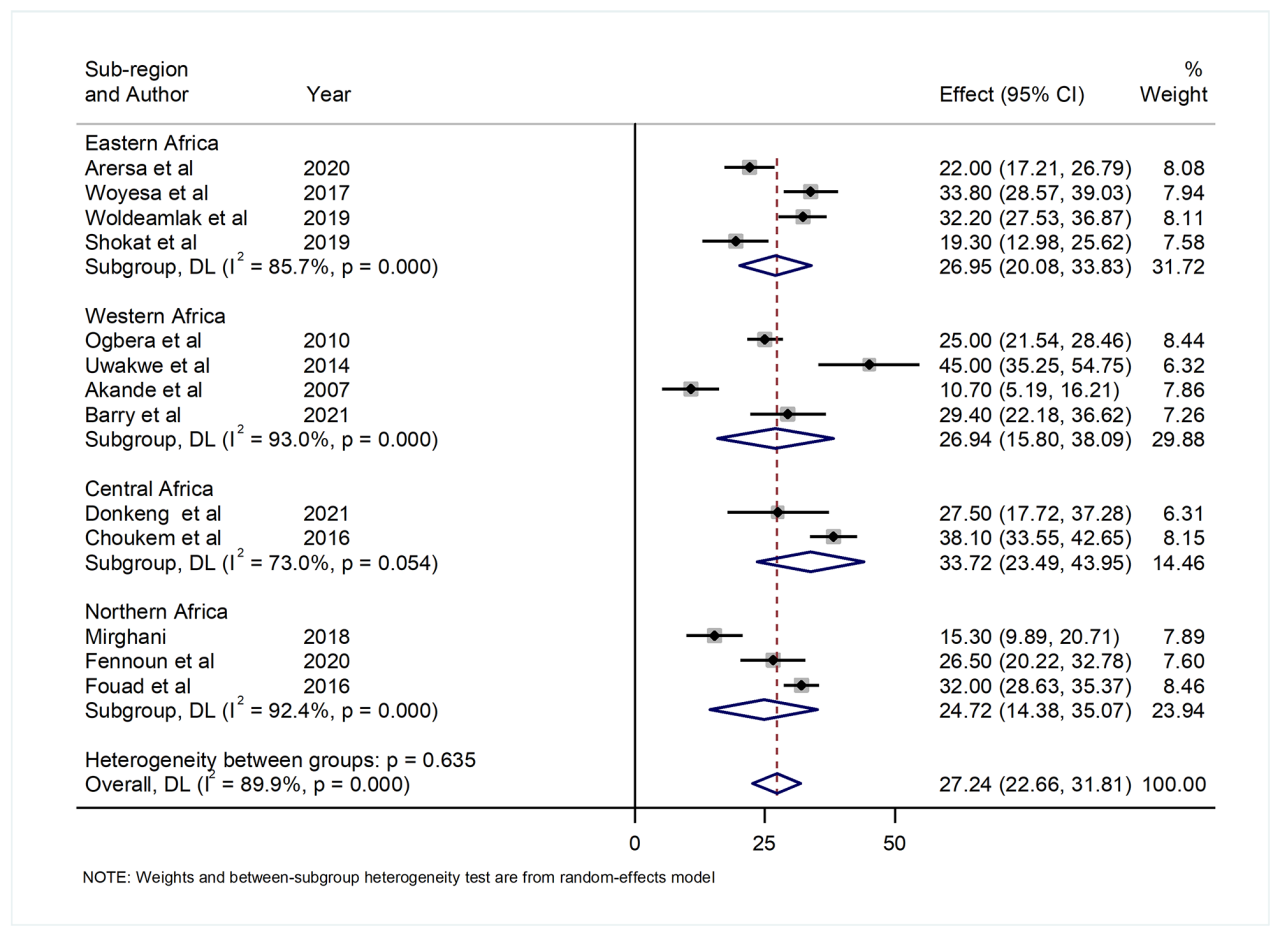
Forest plot showing the pooled prevalence of hyperuricemia by sub-region

The subgroup analysis by sub-region indicated that the pooled prevalence of hyperuricemia among T2DM patients in Africa was highest in Central Africa (33.72% (95% CI: 23.49, 43.95)), followed by East Africa (26.95% (95% CI: 20.08, 38.83)), West Africa (26.94% (95% CI: 15.80, 38.09)), and North Africa (24.72% (95% CI: 14.38, 35.07)). A high level of heterogeneity was seen in all sub-regions with I^2^ of 73%, 85.7%, 93%, and 92.4%, respectively (Fig. 3).

#### Hyperuricemia by sex of the study participants

**Fig. 4:**
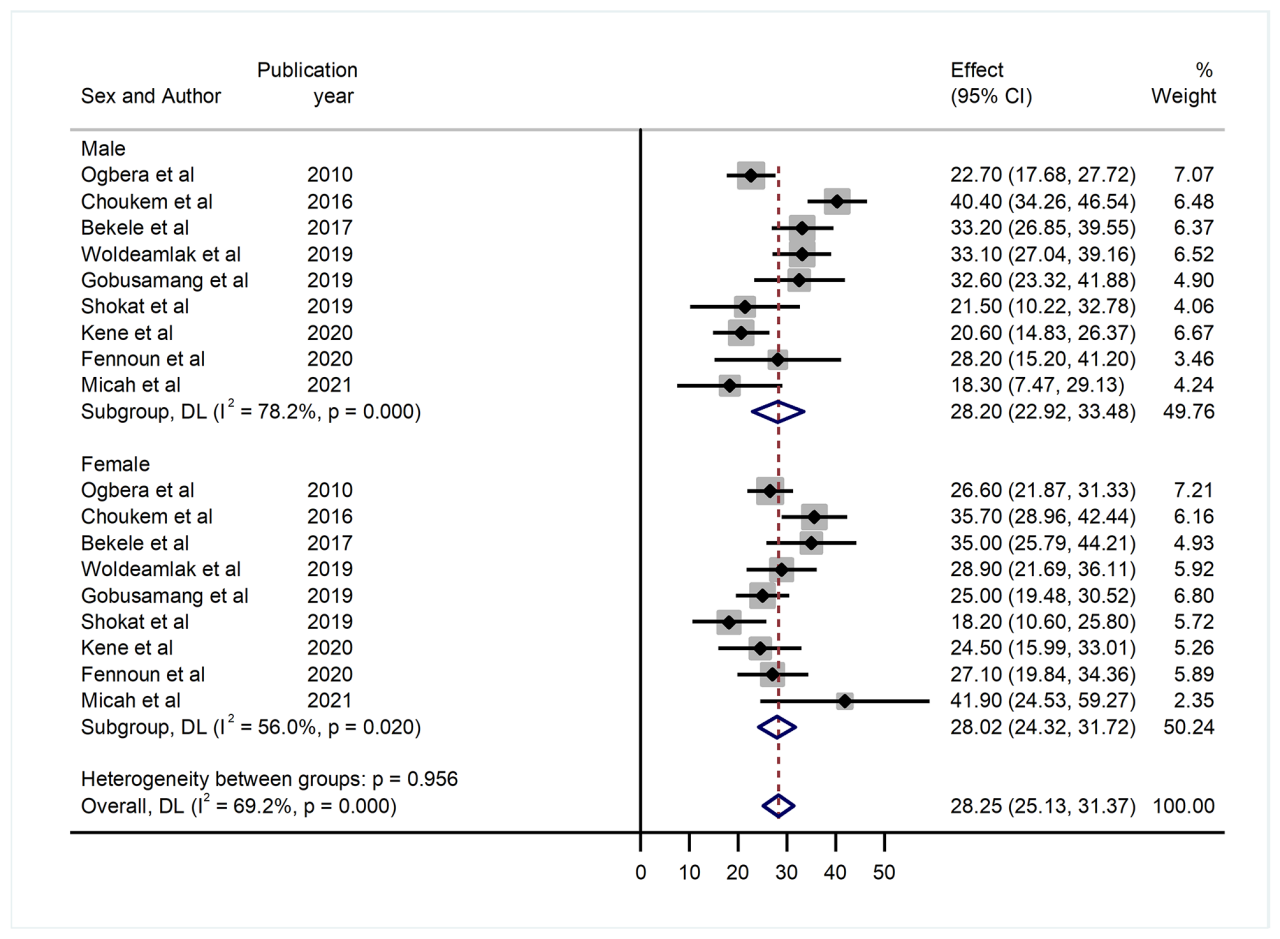
Forest plot showing the pooled prevalence of hyperuricemia by sex of the study participants

In addition, results from subgroup analysis by sex of the study participants, the pooled prevalence of hyperuricemia among female and male patients with T2DM were 28.02% (95% CI: 22.92, 33.48) and 28.20% (95% CI: 22.92, 33.48) respectively. A high level of heterogeneity was observed in both cases with I^2^ of 56.0% and 78.2%, respectively (Fig. 4).

#### Hyperuricemia by country

**Fig. 5:**
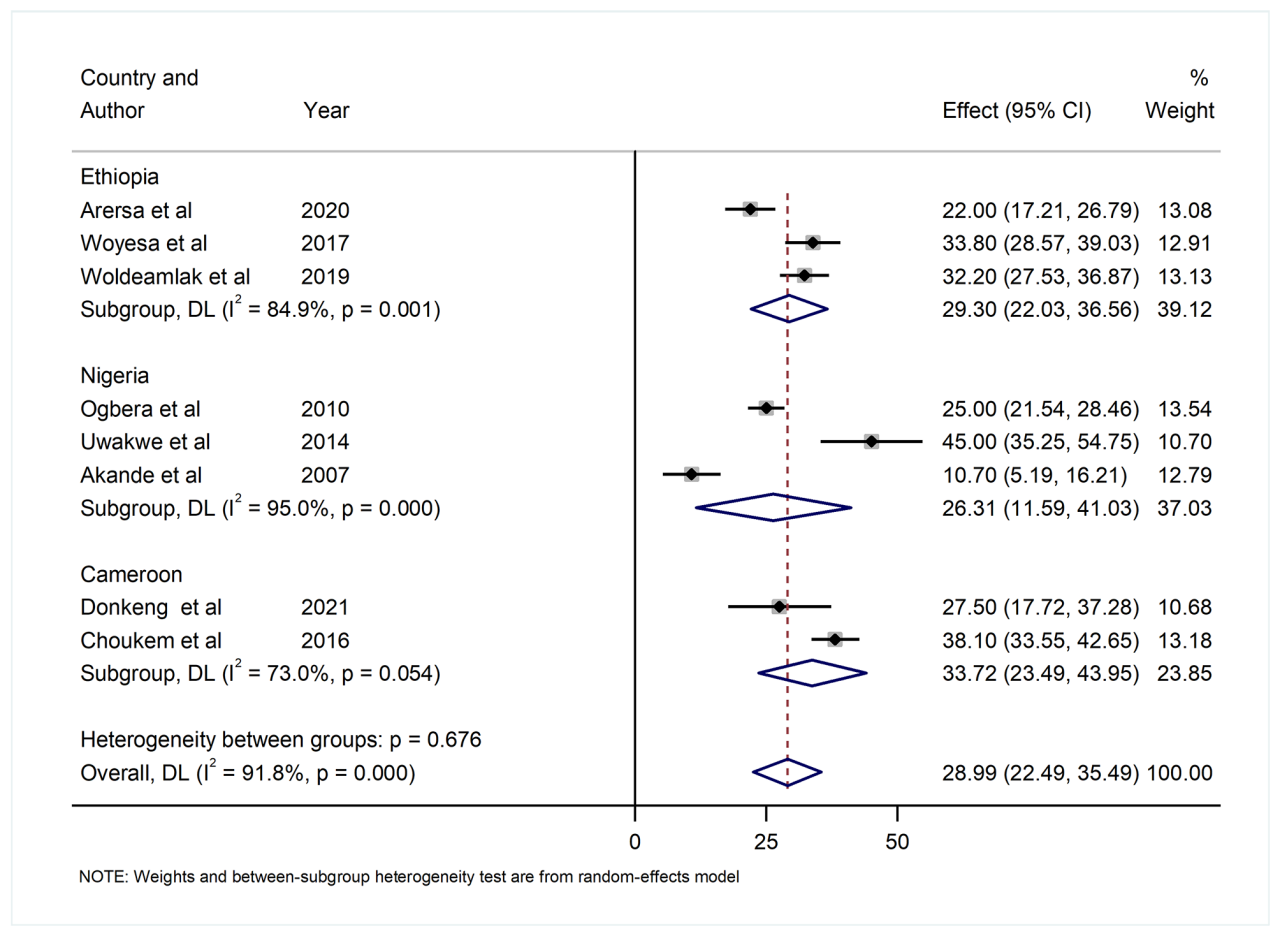
Forest plot showing the pooled prevalence of hyperuricemia by country

Sub-group analysis by country was done for Ethiopia, Nigeria, and Cameroon. The pooled prevalence of hyperuricemia was highest in Cameroon (33.72% (95% CI: 23.49, 43.95)), followed by Ethiopia (29.30% (95% CI: 22.03, 36.56)), and Nigeria (26.31% (95% CI: 11.59, 41.03)). There was a high level of heterogeneity with I^2^ of 73%, 84.9%, and 95%, respectively (Fig. 5).

#### Hyperuricemia by publication year

**Fig. 6:**
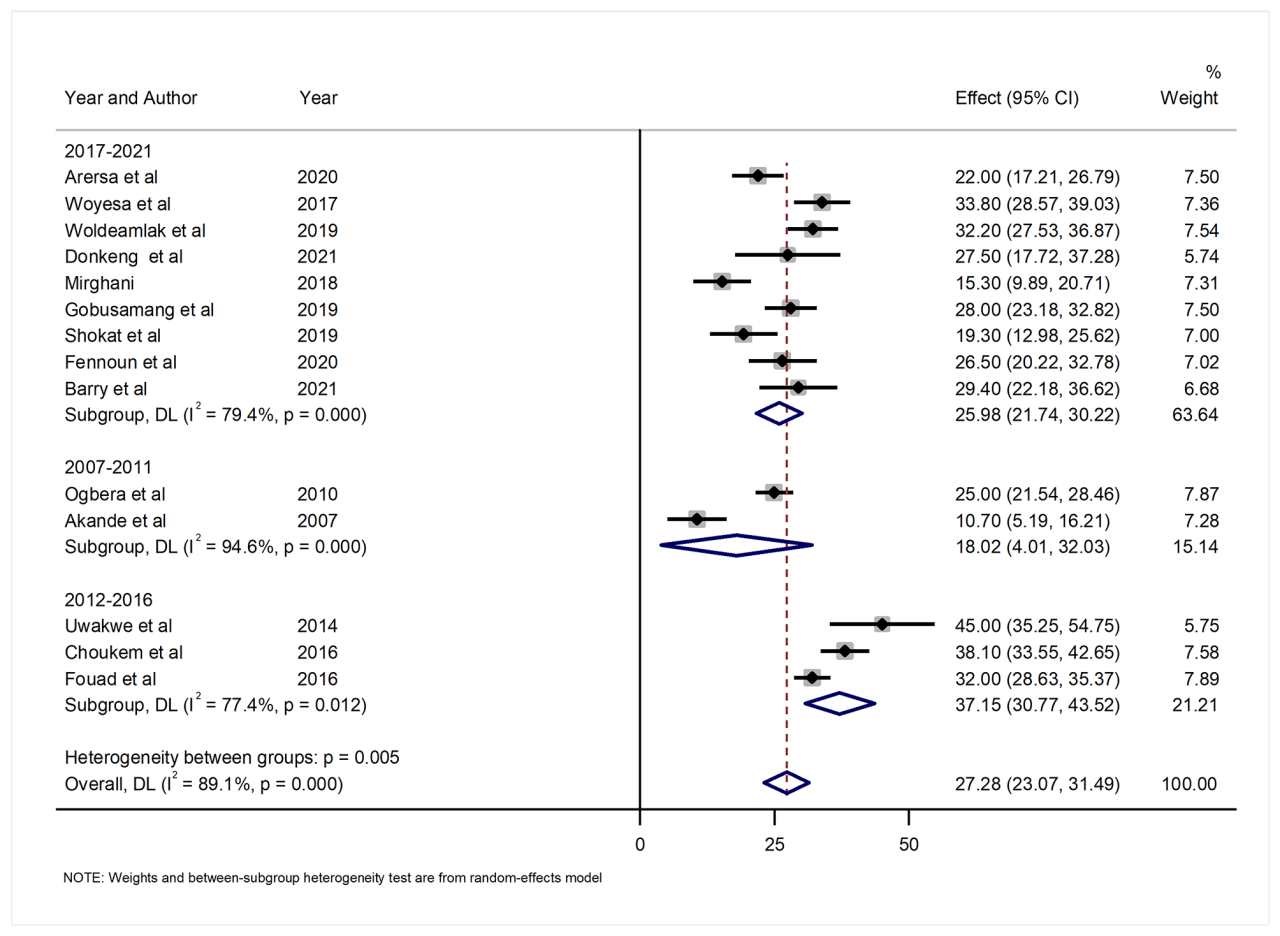
Forest plot showing the pooled prevalence of hyperuricemia by year of publication

Relating to the sub-group analysis by publication year, the results indicated that the highest pooled prevalence of hyperuricemia was observed in 2012–2016 (37.15% (95% CI: 30.77, 43.52)), and the lowest (18.02% (95% CI: 4.01, 32.03)) in 2007–2011. There was a high level of heterogeneity with I^2^ of 94.6%, 77.4%, and 79.4%, respectively (Fig. 6).

#### Publication bias

**Fig. 7:**
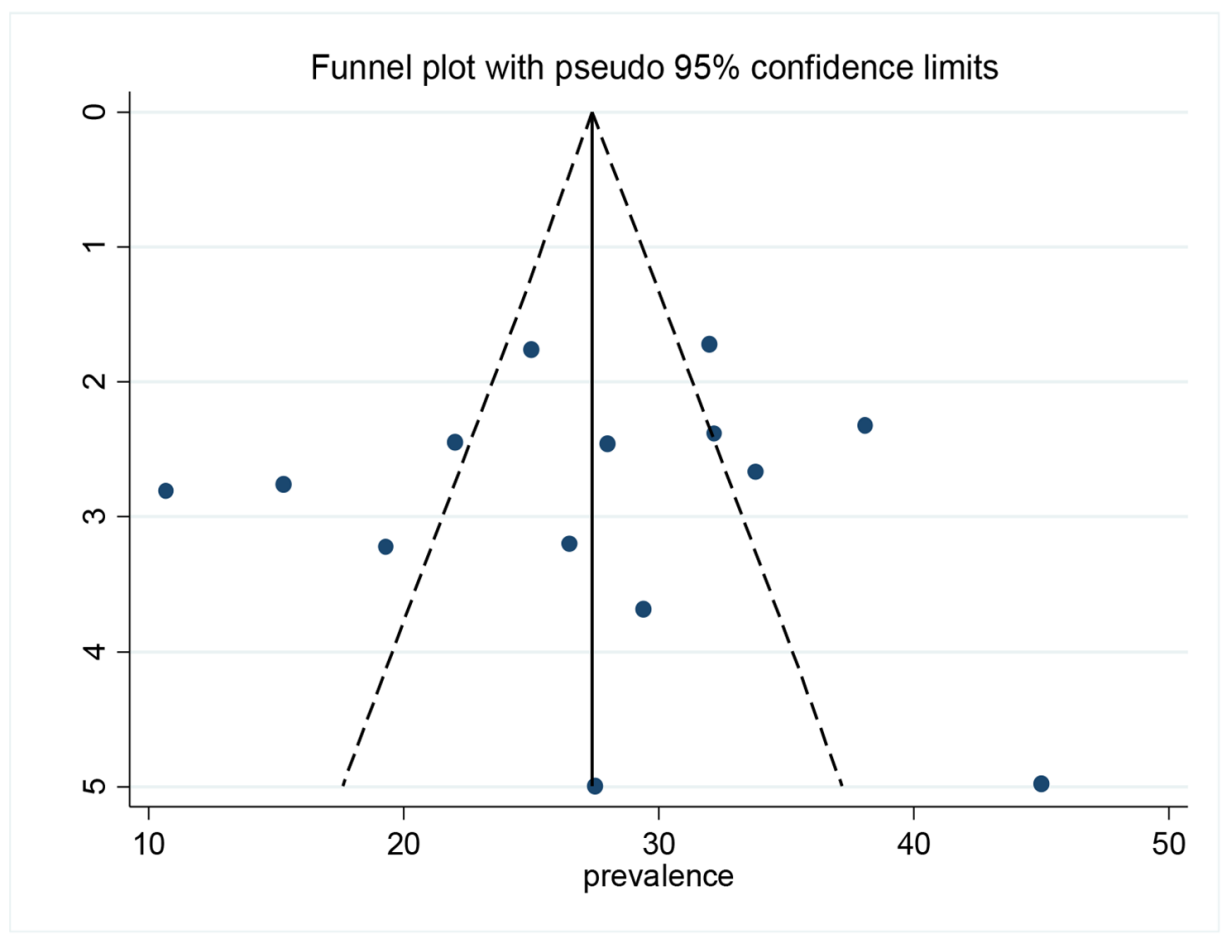
Funnel plot on the prevalence of hyperuricemia among T2DM patients in Africa

Symmetry of the funnel plot (Fig. 7) and the egger’s test statistics with p-value 0.83 confirm the absence of publication bias.

#### Sensitivity analysis

Sensitivity analysis was performed to evaluate the effect of individual studies on the pooled estimated. When individual study was omitted, the pooled prevalence obtained was within the 95% CI of the overall pooled prevalence. This confirms the absence of single study impact on the overall pooled effect size (Table 2).

**Table 2:** Sensitivity analysis of the prevalence of hyperuricemia among type 2 diabetes mellitus patients in Africa

Study omitted	Estimate	95% CI
Arersa et al. (2020)	27.86	26.45–29.27
Woyesa et al. (2017)	26.94	25.54–28.34
Woldeamlak et al. (2019)	26.96	25.54–28.37
Ogbera et al. (2010)	27.83	26.36–29.30
Uwakwe et al. (2014)	27.05	25.69–28.42
Akande et al. (2007)	28.47	27.07–29.86
Donkeng et *al* (2021)	27.39	26.03–28.76
Choukem et al. (2016)	26.36	24.94–27.77
Mirghani (2018)	28.20	26.81–29.60
Gobusamang et al. (2019)	27.34	25.94–28.75
Shokat et al. (2019)	27.79	26.40-29.17
Fennoun et al. (2020)	27.44	26.06–28.82
Barry *et al. (*2021)	27.32	25.95–28.70
Fouad et al. (2016)	26.51	25.04–27.99
Combined	27.40	26.04–28.75

## Discussion

This systematic review and meta-analysis was directed to estimate the pooled prevalence of hyperuricemia among T2DM patients in Africa. T2DM is a huge global public health concern and it has a lot of modifiable and non-modifiable factors [5]. One of the contributing factors to the increase in T2DM is obesity. Due to this, there are some similarities in the therapeutic options currently available for managing and treating obesity and T2DM, including lifestyle changes, medication, different recently developed medical devices, and increasingly common and sophisticated bariatric surgeries [41]. Long-lasting maintained weight loss is possible with bariatric surgery. Additionally, it is crucial to note that many obese people have nutritional deficiencies before surgery, the most significant of which are magnesium and iron deficiency [42]. Following bariatric surgery, other than ghrelin, glucagon-like peptide-1, and peptide YY hormones, weight recovery may be influenced by some of the same factors that contributed to the initial rapid weight loss [43].

Hyperuricemia contributes to the different pathological mechanisms of diabetes and its chronic complications. Mechanisms include inhibiting insulin pathway, endothelial dysfunction, inflammation, oxidative stress, thrombus formation, and activation of the renin-angiotensin-aldosterone system [44]. A meta-analysis of 9 studies including 20,891 T2DM patients also indicated that it is a significant predictor of vascular complications and cardiovascular mortality in patients with T2DM [45].

In this review, the pooled prevalence of hyperuricemia among T2DM patients in Africa was 27.28% (95% CI: 23.07, 31.49). The possible cause of hyperuricemia in T2DM patients might be due to decreased excretion of UA, consequences from the diminished effect of insulin [46], and increased purine production results from increased activity of the hexose monophosphate pathway shunt [47, 48], which occurs during insulin resistance and/or hyperinsulinemia. On the other hand, the build-up of citric acid leads to inhibition of the enzyme phosphofructokinase, which redirects the cycle to the formation of 6 phospho-gluconate and the formation of purine nucleotides, thereby increasing uric acid levels during the expansion of diabetes [49].

The finding of this study was lower than a study conducted in China, which reported that 32.6% of T2DM patients had hyperuricemia [50]. However, this finding was higher than studies reporting hyperuricemia among the general population in China pooled prevalence of 13.3% (95% CI: 11.9%, 14.6%) [51], Australia 16.6% [52], and China 13.5% [53]. This difference might be due to sociocultural, environmental, and economic factors, differences in the cut of values used to define hyperuricemia, study design, heterogeneity of study participants, genetic pattern, and knowledge about risk factors.

The high level of heterogeneity (I^2^ = 89.1%) observed in this study might be due to numerous reasons. It might be due to prevalence differences in sub-regions of Africa, country, year of publication, and sex of study participants. As the result, we considered post-hoc subgroup analyses by different features such as sub-regions of Africa, country, year of publication, and sex of participants. According to subgroup analysis by sub-regions of Africa, the random effect model revealed that the highest pooled prevalence of hyperuricemia was reported in Central Africa (33.72%: 95% CI; 23.49, 43.95), and the lowest was in North Africa (24.72%: 95% CI; 14.38, 35.07). In addition, subgroup analysis by country indicated that the highest pooled prevalence of hyperuricemia was reported in Cameroon (33.72%; 95% CI; 23.49, 43.95), and the lowest was in Nigeria (26.31%: 95% CI; 11.59, 41.03). The possible clarification for these inconsistencies might be related to the differences in the study participants, glycemic control status, cultural differences, cut-off values, number of studies, sample size, and genetic pattern.

Based on results from subgroup analysis by sex of participants showed that the prevalence of hyperuricemia was comparable among females and male T2DM patients were 28.02% (95% CI: 22.92, 33.48) and 28.20% (95% CI: 22.92, 33.48) respectively. Moreover, the subgroup analysis by year of publication revealed that the pooled prevalence of hyperuricemia in 2012–2016 (37.15%: 95% CI; 30.77, 43.52) was higher than in 2007–2011 (18.02%: 95% CI; 4.01, 32.03). This difference might be attributable to the above expiations. However, still, subgroup analysis revealed that the presence of high heterogeneity and some differences across groups may not be statistically trustworthy because of the CIs overlap.

Moreover, this review has some strengths and limitations. It allows determining the current and true pooled prevalence of hyperuricemia among T2DM in Africa. We have performed subgroup analysis (sub-regions of Africa, country, year of publication, and sex of the participants), and followed the PRISMA guideline appropriately, which is considered the strength of our study. Moreover, our meta-analysis has limitations, such as the occurrence of significant heterogeneity even after subgroup analysis, only articles published in the English language were included. Hence the results of this meta-analysis had substantial heterogeneity and there was some overlap of CIs in the subgroup analysis. Finally, it was not able to evaluate factors associated with the pooled prevalence of hyperuricemia.

## Conclusion

This systematic review and meta-analysis showed a high prevalence of hyperuricemia among T2DM patients. It sounds that regular screening and diagnosis of hyperuricemia required in T2DM patients for preventing its pathological effects and contribution to chronic complications of diabetes. Subsequent follow-up is also essential for reducing diabetes-associated mortality and improving the quality of life for an individual living with type 2 diabetes.

## Electronic supplementary material

Below is the link to the electronic supplementary material.

Supplementary Material 1

## Data Availability

All necessary data for this systematic review and meta-analysis are available within the manuscript and its supporting information.

## Abbreviations

T2DM: Type 2 Diabetes Mellitus
UA: Uric Acid
